# Supplementary material for: Patterns of Intron Gain and Loss in Fungi
Source: PLoS Biol. 2004 Nov 30;2(12):e422. doi: 10.1371/journal.pbio.0020422 (PMC532390; doi:10.1371/journal.pbio.0020422)
Supplement: Table S1 — Also available at http://genes.mit.edu/NielsenEtAl/. (4.3 MB ZIP). [file pbio.0020422.st001.zip › NielsenEtAl/html/1273.html]

AN2475.1.NCU00821.1.MG09852.1.FG05839.1


```
 CLUSTAL W (1.82) Multiple Sequence Alignments - Introns Inserted


Sequence 1: MG09852.1	568 aa
Sequence 2: FG05839.1	540 aa
Sequence 3: NCU00821.1	546 aa
Sequence 4: AN2475.1	490 aa
Alignment Length: 577 aa
Number Identitical Residues: 234 aa
Alignment Score (without introns) 12566


MG09852.1 	MAPPKFMGMSGRPLSTAVSTVATTGFLLFGYDQG2VMSGIISAPAFNKFFPETDRNPTMQ
NCU00821.1	MAPPKFLGLSGRPLSLAVSTVATTGFLLFGYDQG2VMSGIITAPAFNNFFTPTKDNSTMQ
FG05839.1 	-MPPTFAGLTGKKLSLAISTVATTGFLLFGYDQG~VMSGIIDADPFHDYFPET-KNSTMQ
AN2475.1  	----------------------------------~-MSGIISDVAFNDVFTATKDDATMQ
          	                                    *****   .*:. *. *. :.***

MG09852.1 	GFVTAIYEIGCLLGAMFILAVGDILGRRRAMILGALIMGLGVVIQIVTIKPSPSPIAQFI
NCU00821.1	GLITAIYEIGCLIGAMFVLWTGDLLGRRRNIMVGAFIMALGVIIQVTCQAG-SNPFAQLF
FG05839.1 	GFVTAIYEIGCLLGAMFILWIGDLLGRRRAMILGGWIMILGVIIQITAMKG-HSALAQLI
AN2475.1  	ALVTAVYELGCLAGAVYALMFGDRMGRRWMILSGAAVMILGVIIQVTSFPG-HIPLLQFF
          	.::**:**:*** **:: *  ** :***  :: *. :* ***:**:.       .: *::

MG09852.1 	IGRTIMGVGNGINT~STIPTYQAECSQTSNRGLLLCIQG~GMIAFGTVIAYWIDYGASYG
NCU00821.1	VGRVVMGIGNGMNT~STIPTYQ----------------G1GVIAFGTLIAYWIDYGASYG
FG05839.1 	VGRTITGVGNGINT~STIPTYQAECSTTSNRGLLICIEG~GIIAFGTLIAYWIDYGCSYG
AN2475.1  	IGRVITGIGNGMNT2STIPTYQAECSKTSNRGLLICIEG~GIIAIGTAIAYWIDFGSHYG
          	:**.: *:***:** *******:..: ::. .   . .* *:**:** ******:*. **

MG09852.1 	PDDLVWRFPIAFQIVFALFISIPMCFLPESPRWLLTHQRVPEADRVIAALRGYEIDGQET
NCU00821.1	PDDLVWRFPIAFQLLFAIFICVPMFYLPESPRWLLSHGRTQEADKVIAALRGYEIDGPET
FG05839.1 	PQDLTWRFPIAFQVVFGLVVCLGMVWLPESPRWLLTHDRHEEAEKVIAAIRGFEIDSDET
AN2475.1  	PPDLVWRFPIAFQIIFGIIIVVGMWFLPDSPRHLIAKGRIEEGEYVLAALNGCEVHDHET
          	* **.********::*.:.: : * :**:*** *::: *  *.: *:**:.* *:.. **

MG09852.1 	ILERDLILDSIKASGFSGQKSTPVKALFTGGPTQHLRRMLLGSGSQLMQQIGGCNAVIYY
NCU00821.1	IQERNLIVDSLRASGGFGQKSTPFKALFTGGKTQHFRRLLLGSSSQFMQQVGGCNAVIYY
FG05839.1 	RAERDRVVDSIRASGFAAQKSTPVKALFTGGKTQHFRRMLLGAGSQFMQQVGGCNAVIYY
AN2475.1  	QIQKQLTLDSLRASGALG-KKTSFRDLLTGGPSQHLRRMLVGSSSQVFQQLGGCNAVIYY
          	  :::  :**::***  . *.*..: *:*** :**:**:*:*:.**.:**:*********

MG09852.1 	FPILFQESIGETHEMALLLGGVNMIVYAIFATTSWFVIERAGRRKLFLIGSIGQCLSMVI
NCU00821.1	FPILFQDSIGESHNMSMLLGGINMIVYSIFATVSWFAIERVGRRRLFLIGTVGQMLSMVI
FG05839.1 	FPILCTDIFGD-KNFALLLGGVNMIVYSIFATSSWFLIERVGRRKLLLIGTAGQMLSMFL
AN2475.1  	LPVLLKSNLHQDNDQALLIGGINMIVYAVFATFSWFFIERIGRRKLFLGGSMVQTTAMII
          	:*:*  . : : :: ::*:**:*****::*** *** *** ***:*:* *:  *  :*.:

MG09852.1 	TFACLIPG-------TTSAAKGAAVGLFTYIAFFGATWLPLPWLYPAEVNPIKTRAKANA
NCU00821.1	VFACLIPD-------DPMKARGAAVGLFTYIAFFGATWLPLPWLYPAEVNPIRTRGKANA
FG05839.1 	TMGFLIPGGSDPGNNAPQISKGAIAGLFTYIASFGATWLPLPWLYPAEINPLKTRGKANA
AN2475.1  	TFACLIPG-------DEQSSKGAVFGLFLYMAAFGATWLPLPWLYPAELSPVRTRAKANA
          	.:. ***.           ::**  *** *:* ***************:.*::**.****

MG09852.1 	VSTCTNWLFNFLVVMVVPIMIANIGWGTYLFFAIMNALFIPIIYFFFPE~TKRRSLEEID
NCU00821.1	VSTCSNWMFNFLIVMVTPIMVDKIGWGTYLFFAVMNGCFLPIIYFFYPE~TANRSLEEID
FG05839.1 	TSTCTNWLFNFVIVMIVPIMISNIHWGTYLFFGCANATFFPILYWFYPE0TANRSLEEID
AN2475.1  	VSTCNNWLFNFAVVMFTPPMVAAIGWGTYLFFAAWNAFFIPVIWFFYPE~TANRSLEEID
          	.***.**:*** :**..* *:  * *******.  *. *:*::::*:** * .*******

MG09852.1 	VIFAKGFSEKTSYVTAAKELPYLAPEEVEGESRKYGLISASGQDATGAGNDATRRNSEKP
NCU00821.1	IIFAKGFVENMSYVTAAKELPHLTAEEIESYANKYGLVDRDSNGEGG-----NRHDEEKT
FG05839.1 	IIFAKGFVEQISYVKAAKELPFLTQEEVEREAIRLGLVDEAGRGG----------MMEKP
AN2475.1  	LIFAKGYHEKISYVRASHELPKLSDDEIEAEAAKYQYSPET----------------EAE
          	:*****: *: *** *::*** *: :*:*  : :                       *  

MG09852.1 	YEDPRQNSIWGNGSDEESQTRNTSVAE--PSGANTNTTKHG
NCU00821.1	RDRPDQSDSDSPAHVEIDVVDEHGVESGFGDGINTKETR--
FG05839.1 	GEE------------------SGTVRD--ESGFNSEKS---
AN2475.1  	VEK-------------------GHVAH---PGSTSE-----
          	 :                      *      * .::
```
